# The scope of antimicrobial resistance in residential aged care facilities determined through analysis of *Escherichia coli* and the total wastewater resistome

**DOI:** 10.1128/spectrum.00731-23

**Published:** 2023-10-03

**Authors:** Sylvia A. Sapula, Anteneh Amsalu, Jon J. Whittall, Bradley J. Hart, Naomi L. Siderius, Lynn Nguyen, Cobus Gerber, John Turnidge, Henrietta Venter

**Affiliations:** 1 Health and Biomedical Innovation, UniSA Clinical and Health Sciences, University of South Australia, Adelaide, South Australia, Australia; 2 Department of Medical Microbiology, University of Gondar, Gondar, Ethiopia; 3 Adelaide Medical School, University of Adelaide, Adelaide, South Australia, Australia; Columbia University, New York, New York, USA

**Keywords:** antimicrobial resistance, wastewater-based epidemiology, *Escherichia coli*, multidrug resistance, metagenomics

## Abstract

High and often inappropriate antibiotic use has been documented for residential aged care facilities (RACFs). As a result, RACFs represent selective environments favoring the development of antimicrobial resistance (AMR). However, surveillance of the prevalence and scope of AMR in RACFs is limited. Here, wastewater-based epidemiology encompassing culture-based methods in combination with whole genome sequencing and metagenomics allowed for the in-depth analysis of the prevalence of antimicrobial-resistant *Escherichia coli* in two RACFs and one retirement facility. Wastewater was collected at five different time points over 18 months. From these, *E. coli* were isolated and assessed for phenotypic and genotypic resistance. The antimicrobial resistome of each wastewater sample was also determined. A comparison of facilities revealed a higher prevalence of AMR and multidrug-resistant *E. coli* observed in one RACF. The international high-risk *E. coli* clone, ST131, carrying CTX-M-like extended-spectrum beta-lactamases was exclusively isolated from this facility. A high proportion of these isolates were classified as belonging to phylogroups B2 and D, associated with virulent extra-intestinal *E. coli* infections. The resistome of this facility also revealed a higher prevalence of mobile resistance genes such as *sul1* and *sul2*, conferring sulfamethoxazole resistance, the plasmid-mediated quinolone-resistance gene *qnrS*, and *qacEdelta1* that confers biocide resistance. High fluoroquinolone resistance rates were observed in all three sample sites despite measures in place limiting the use of this class of antibiotics. The findings of this study illustrate that RACFs are highly selective environments that require measures to limit AMR development, potentially through antimicrobial stewardship.

**IMPORTANCE:**

Antimicrobial resistance (AMR) is a global threat that imposes a heavy burden on our health and economy. Residential aged care facilities (RACFs), where frequent inappropriate antibiotic use creates a selective environment that promotes the development of bacterial resistance, significantly contribute to this problem. We used wastewater-based epidemiology to provide a holistic whole-facility assessment and comparison of antimicrobial resistance in two RACFs and a retirement village. Resistant *Escherichia coli*, a common and oftentimes problematic pathogen within RACFs, was isolated from the wastewater, and the phenotypic and genotypic AMR was determined for all isolates. We observed a high prevalence of an international high-risk clone, carrying an extended-spectrum beta-lactamase in one facility. Analysis of the entire resistome also revealed a greater number of mobile resistance genes in this facility. Finally, both facilities displayed high fluoroquinolone resistance rates—a worrying trend seen globally despite measures in place aimed at limiting their use.

## INTRODUCTION

Antimicrobial resistance (AMR) is an urgent and global threat (1), with recent reports estimating that approximately 4.95 million AMR-associated deaths occurred in 2019 (2). Continual AMR development is also speculated to add to the global economic burden, as projections indicate that 300 million people will lose their lives to AMR over the next 35 years, resulting with a loss of 60 to 100 trillion USD worth of economic output (1). In addition to increased mortality rates, AMR can lead to increased duration of illness particularly for those who are immunocompromised (3, 4). In the elderly population, where individuals are at an increased risk of incidence and severity of bacterial infections, AMR bacteria contribute significantly to the burden of infection, with infectious diseases accounting for one-third of all deaths in those aged 65 years and over (5 – 7).

A significant contributor to the development and spread of AMR is the overuse and misuse of antibiotics, which promotes the selection of AMR bacteria (8 – 11). Residential aged care facilities (RACFs) exemplify the overuse and frequent inappropriate use of antibiotics (12 – 14). This, coupled with the vulnerability of the residents resulting from comorbidities, a compromised immune system, close living proximities, hospital visits, and frequent healthcare worker contact, facilitates the spread of resistant bacteria and promotes a high infection burden among the residents (15 – 17). As such, RACFs and residents themselves have been identified as important reservoirs for the development of emerging AMR and multidrug-resistant (MDR) bacteria (18).

Surveillance has often been limited to healthcare setting such as hospitals, where pathogens isolated from clinical infections are reported (19, 20). Surveillance studies of these in wastewater from RACFs are scant (21). Studies assessing AMR in RACFs are resident focused and, although vital, are fraught with limitations regarding the number of residents included in each study and, as such, do not always represent the facility as a whole. Nonetheless, such studies have reported a high prevalence of MDR extended-spectrum beta-lactamase (ESBL)-producing *Escherichia coli* (22, 23). A point prevalence survey of three Melbourne-based RACFs revealed that 27% of residents were colonized with ESBL-producing *E. coli*, which were clonal in nature (22). An increasing prevalence of ESBL-producing *E. coli* has also been observed in aged care facilities in Germany (24, 25), Japan (26), and the Netherlands (27). ESBL-producing ST131 *E. coli* were also observed in aged care facilities in Germany (24) and Portugal (28). This clonal type, also referred to as a worldwide pandemic clone (29), has spread rapidly throughout different parts of the world since its discovery in 2008 (30). In addition to ESBL expressing ST131, a UK study has also found fluoroquinolone-resistant but ESBL-lacking ST131 strains carrying the plasmid-mediated *aac(6′)-Ib-cr* gene, which reduced susceptibility to ciprofloxacin (31), suggesting a split and continual emergence of ST131. In Australia, rates of *E. coli* found to be resistant to beta-lactams such as ceftriaxone and fluoroquinolones such as ciprofloxacin continue to increase, despite restrictions being in place to limit access to these agents (32). The prevalence of these underscores the importance of surveillance studies in RACF, with the use of wastewater-based surveillance offering an insight into the facility as whole.

Therefore, the aim of this study was to utilize wastewater-based epidemiology, consisting of microbial culturing, whole genome sequencing (WGS), and metagenomics to investigate AMR within two Adelaide RACFs. The use of wastewater in this thorough investigation has circumvented one of the most common limitations in studies of AMR in RACFs—low recruitment numbers of participants—and allowed for a holistic approach to the assessment of AMR in entire RACFs. This study demonstrates the importance of surveillance studies focusing on AMR within RACFs. Housing a vulnerable older population, the development of bacterial resistance within these facilities contributes to a growing burden of infection for which treatment may become more limited. As an aging population, with one in six estimated to be aged over 60 years old by 2030 (33), the development of AMR within RACFs, as such, is area of great concern. This study exemplifies a novel approach to the assessment of AMR within RACFs, as the use of wastewater, microbial culturing, WGS, and metagenomics to assess the development of microbial resistance has not previously been undertaken in this setting. The inclusion of one facility which has implemented an antimicrobial stewardship (AMS) program and another which has not also allowed for an assessment of the effect that an AMS program may have on the prevalence of AMR bacteria within a facility.

## MATERIALS AND METHODS

### Sampling

Wastewater was sampled from two RACFs (Facility 1 with 170 beds and Facility 2 with 70 beds) and one retirement village (38 apartments with residents of similar age to those from the RACFs) in Adelaide. All sites are managed by the same service provider and are located within a 20-km radius of each other. Of the two RACFs, Facility 2 has had an AMS program (File S1) implemented for 3 years prior to this study, while Facility 1 does not have an AMS program implemented. As both aged care facilities are managed by the same care provider, they are comparable in the amenities and care provided. Both facilities have long-term residents in addition to respite care.

Wastewater samples were collected at approximately 3-month intervals at five different time points from October 2019 to February 2021. Grab samples of approximately 200 mL were collected every hour over a 10-hour period, with collection starting at 7 a.m. to capture the morning routine. These were collected from an access point which captured all of wastewater from the RACF before it flowed into the main sewage system. Samples were then stored on ice and pooled for analysis. Sampled wastewater was transported to the laboratory on ice, stored at 4°C, and analyzed on the day of collection.

### Isolation of *Escherichia coli*

AMR *E. coli* isolates were screened on selective and differential plates. For isolation of *E. coli* from wastewater, 100-µL wastewater (neat and 1:10 dilution) was plated onto selective and differential plates. Presumptive identification of *E. coli* was determined using two sets of Brilliance *E. coli*/coliform (CM0956, Oxoid, Australia) plates supplemented with either 1 mg/L ceftazidime or 0.5 mg/L of ciprofloxacin and Tergitol 7 (CM0793, Oxoid) plates supplemented with 0.5 mg/L ciprofloxacin. Cultures were incubated at 37°C for 18 hours. Following colony purification, identification was verified by matrix-assisted laser desorption/ionization time-of-flight mass spectrometry (Bruker Daltonik GmbH, Bremen, Germany). Confirmed *E. coli* isolates were stored at −80°C in Tryptone Soya broth (CM0129, Oxoid) supplemented with 20% (vol/vol) glycerol.

### Antimicrobial susceptibility testing

Antibiotics assessed in this study included cefepime, ceftazidime, piperacillin-tazobactam, gentamicin, meropenem, trimethoprim-sulfamethoxazole, colistin (ChemSupply, Australia), and ciprofloxacin (Sigma-Aldrich, Australia). The minimum inhibitory concentration (MIC) of each antibiotic assessed in this study was determined using the broth dilution method as described by the ISO standard (34 – 36). Briefly, a 96-well microtiter plate was prepared with twofold serial dilutions of the antimicrobial agent. Cultures at an OD_600_ of 0.0025 nm were used as an inoculum using Mueller-Hinton broth as a growth medium. *E. coli* ATCC 25922 was used as a quality control strain. The 96-well microtiter plates were incubated at 37°C with 120 rpm shaking for 16 hours. Resistance/susceptibility was determined by measuring bacterial growth by optical density at 600 nm using a Thermo Fisher Multiskan FC photometer (Thermo Fisher, Australia) using *E. coli* ATCC 25922 for quality control. Epidemiological cutoff values, as reported by the European Committee on Antimicrobial Susceptibility Testing (https://mic.eucast.org/search/), were used to distinguish wild-type from non-wild-type isolates. In this manuscript, the terms “susceptible” and “resistant” are used to refer to “wild type” and “non-wild type.” Moreover, resistance to at least one antimicrobial agent in ≥3 antimicrobial categories were considered as MDR (37). To ensure assessment was not carried out on duplicate *E. coli* isolates, the resistance profile of all *E. coli* was evaluated. Isolates observed to have the same resistance profile and that were isolated from the same site at the same time point were subsequently eliminated from further assessment.

### DNA extraction

#### Genomic extraction for whole genome sequencing

Following colony purification, a single colony was incubated in Luria-Bertani (LB) broth and grown at 37°C overnight. Bacterial cells were obtained by centrifugation (5,000 × *g*, 5 min) of 5 mL of culture broth. Genomic DNA was extracted from the pellet using the MN NucleoSpin Microbial DNA kit (Machery-Nagel GmbH and Co. KG, Duren, Germany), following manufacturer’s instructions. DNA quality and quantity were assessed using a Cytation5 imaging reader (BioTek instruments, Winooski, Vermont, USA). Extracted genomic DNA was stored at −20°C (35, 38).

#### Genomic extraction for shotgun metagenomic sequencing

Wastewater samples (100 mL) were initially filtered through an 8-µm cellulose nitrate filter to remove debris and then through a 0.2-µm pore size cellulose nitrate filter (both from Sartorius, Goettingen, Germany). Following filtration, genomic DNA was extracted from the membranes using the DNeasy PowerWater kit (Qiagen, Hilden, Germany), following manufacturer’s instructions. DNA extracts were stored at −20°C prior to being analyzed by metagenomic sequencing. DNA quantity and quality were assessed spectrophotometrically using the Cytation5 imaging reader (BioTek Instruments, Winooski, Vermont, USA) and the Take3 MicroVolume plate.

#### Whole genome sequencing and bioinformatic analysis

WGS was performed at SA Pathology (Adelaide, Australia) using the Illumia NextSeq platform. Sequencing libraries were prepared using the Nextera XT DNA library preparation kit (Illumina Inc., USA) as per manufacturer’s instructions. WGS was performed on the Illumina NextSeq 550 platform with the NextSq 500/550 Mid-Output kit v2.5 (300 cycles) (Illumina Inc). Raw 150-bp paired-end reads were used as input data for the TORMES v.1.2 (39) pipeline for the analysis of whole bacterial genomes. This included sequence quality filtering (PRINSEQ v.0.20.4) (40), *de novo* genome assembly (SPAdes v.13.4.1) (41), and annotation (Prokka v1.14.5) (42). *E. coli* multilocus sequencing typing (MLST) profiles were predicted using mlst v2.10 (T. Seemann, https://github.com/tseemann/mlst) which uses the PubMLST database (43). AMR genes were screened using ABRicate (T. Seemann, https://github.com/tseemann/abricate) against the ResFinder (44), Comprehensive Antibiotic Resistance Database (CARD) (45), and ARG-ANNOT (46) databases. Additional software such PointFinder (47) was used to screen for chromosomal point mutations, and PlasmidFinder (48) was used for plasmid replicon screening. Finally, FimTyper (49) and SeroTypeFinder (50) were used to type *E. coli* isolates, while *in silico* phylogroup determination was carried out by ClermonTyping (51). *E. coli* AMR determinants and plasmids were further analyzed using NCBI Blast (52).

A maximum-likelihood phylogenetic tree was constructed to assess *E. coli* clonality. Core genome single nucleotide polymorphisms (SNPs) were identified using CSI phylogeny 1.4 (53). Reads from each *E. coli* isolate were aligned to a reference genome EC958 (GenBank accession no. NZ_HG941718.1). EC958 is an international MDR *E. coli* O25b:H4-ST131 clone which produces the CTX-M-15 ESBL and is fluroquinolone resistant (54). The output file was used to annotate and visualize a phylogenetic tree using the interactive tree of life v.6 (iTOL) tool (55, 56). Unless otherwise noted, default parameters were used for all software tools.

### Metagenomic sequencing and bioinformation analysis

A total of 15 samples were used for shotgun metagenomic sequencing and analysis. Metagenomic sequencing was performed at the SAGC (South Australian Genomics Centre, Adelaide, Australia) and Macrogen, Inc. (Seoul, South Korea), using the Illumina Novaseq S1 platform with 150-bp paired-end reads. Read quality was assessed using FastQC (57). The SqueezeMeta v1.4 (58) pipeline was used for standard metagenomic analysis. SqueezeMeta uses Trimmomatic (59) for filtering and trimming of adapters before assembly using MEGAHIT (60). The resulting contigs were filtered for quality with short contigs removed with Prinseq (40). Gene and rRNA predication were performed utilizing the Prodigal gene prediction software (61), while rRNA sequences were found using barrnap (62). AMR analysis was performed using SqueezeMeta v1.4 (58) by Diamond (63) against version 3.1.4 of the CARD (45). AMR gene abundance was calculated as transcripts per million (TPM) mapped bacterial reads.

### Statistical analysis

Principal component analysis (PCA) was performed in R studio v.1.2.5033 and used here to visually compare the distribution of resistant *E. coli* isolates recovered from wastewater sampled from different sites (facilities). Statistical analysis, bar graphs, and boxplots were generated using Graph Prism v9, with statistical differences between selected AMR gene prevalence and facility assessed by a two-tailed Mann-Whitney U test with results considered statistically significant at *P* < 0.05 and *P* < 0.01 level.

## RESULTS

### Resistance profiles of the *E. coli* isolates reveals a high prevalence of resistance and high-level resistance

Wastewater sampling and analysis were used to evaluate the prevalence and antimicrobial resistance of *E. coli* in two local RACFs and one retirement village. Since fluoroquinolone resistance and ESBL production are of particular concern for *E. coli,* selection of *E. coli* isolates was carried out with selective and differential media supplemented with ciprofloxacin or ceftazidime.

A total of 93 AMR *E. coli* isolates (*n* = 58 from Facility 1, *n* = 27 from Facility 2, and *n* = 8 from a retirement village) were purified from wastewater samples. As expected, due to the selection process used in this study, resistance to ceftazidime and ciprofloxacin was observed for a large percentage of *E. coli* isolates, with 66.7% (*n* = 62/93) and 96.8% (*n* = 90/93) of all isolates shown to be ceftazidime and ciprofloxacin resistant, respectively (Table 1). Interestingly, almost all the isolates were resistant to ciprofloxacin irrespective the media and antibiotic selection/non-selection used for isolation. In addition, a high incidence of trimethoprim-sulfamethoxazole-resistant (*n* = 51/93, 54.8%) and gentamicin-resistant (*n* = 47/93, 50.5%) *E. coli* was also observed, although resistance to these antibiotics were not selected for. Finally, resistance to the last-resort antibiotics meropenem (*n* = 12/9 3, 20.7%) and colistin (*n* = 4/93, 4.3%) was observed, albeit at lower frequencies.

**TABLE 1:** MIC distribution for 93 *E. coli* isolates recovered from wastewater sampled from associated RACFs and a retirement village

	Number of *E. coli* isolates with MIC (mg/L) at:															
Antibiotic	0.008	0.016	0.03	0.06	0.125	0.25	0.5	1	2	4	8	16	32	64	>64	%NWT
FEP	0	0	3	19	16^*b*^	5	3	10	6	1	4	6	8	1	11	54
CAZ	0	0	0	0	4	13	14^*b*^	0	0	1	1	5	18	21	16	67
PTZ	0	0	0	0	0	0	0	9	18	40	10^*b*^	3	2	7	4	17
CIP	0	0	1	2^*b*^	0	6	2	8	9	1	5	1	26	18	14	97
GEN	0	0	0	0	0	0	7	22	17^*b*^	10	2	6	2	6	21	51
MER	8	15	39	19^*b*^	2	2	5	2	1	0	0	0	0	0	0	13
SXT	1	1	16	11	7	4^*b*^	2	0	7	0	1	43^*a*^	0	0	0	55
COL	0	0	0	0	0	0	13	50	26^*b*^	0	0	4	0	0	0	4

^*a*^ MIC of 43 isolates >8 SXT.

^*b*^ EUCAST ECOFF values. FEP, cefepime; CAZ, ceftazidime; PTZ, piperacillin-tazobactam; CIP, ciprofloxacin; GEN, gentamicin; MER, meropenem; SXT, trimethoprim-sulfamethoxazole; COL, colistin; NWT, non-wild-type.

### Analysis of resistance profiles between facilities reveals a difference in the proportion of multidrug-resistant *E. coli* isolates

**Fig 1:**
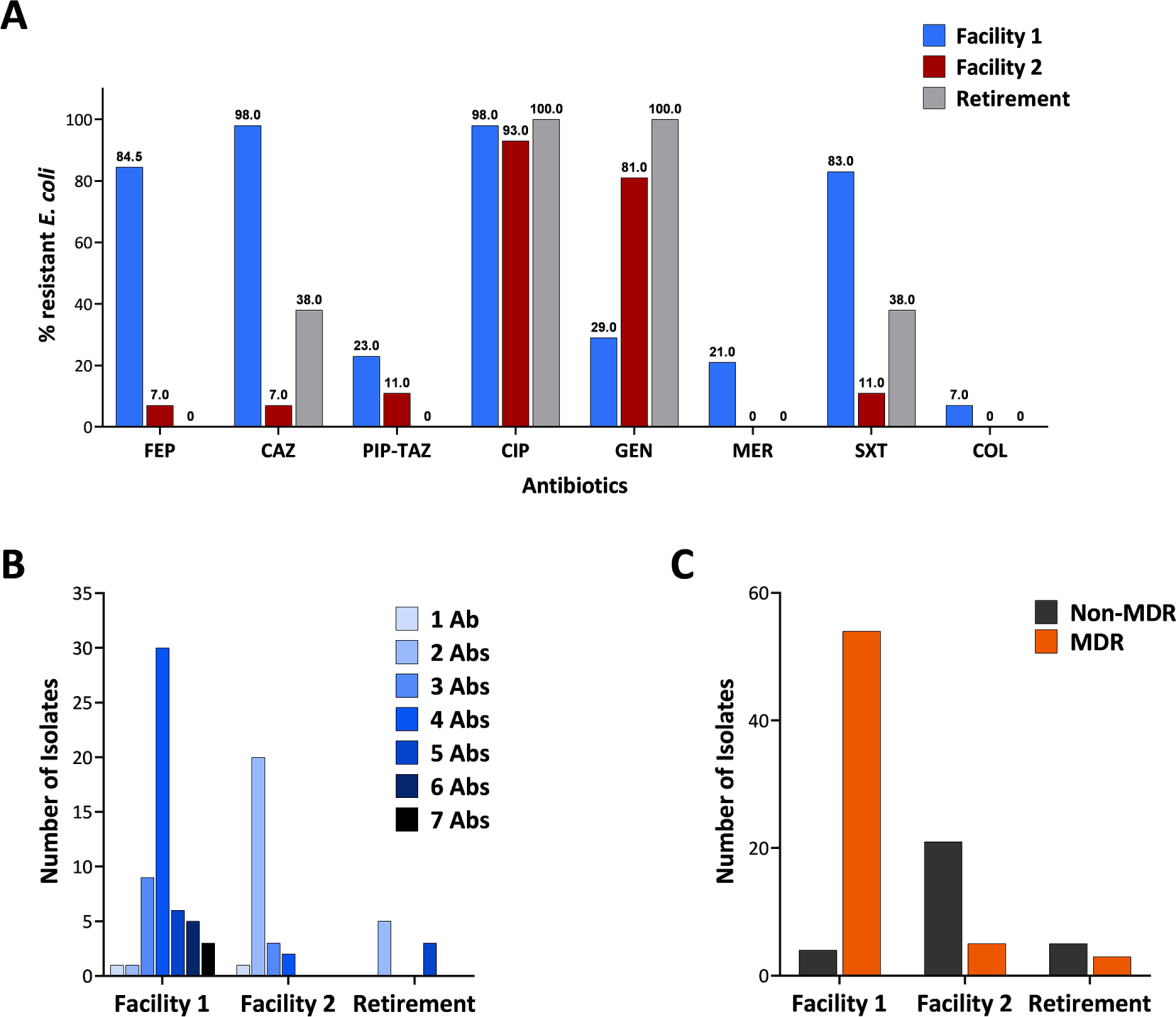
Facility 1 shows the highest prevalence of MDR *E. coli* isolates. (**A**) Percentage of total AMR *E. coli* isolates per facility and retirement village. (**B**) Prevalence of *E. coli* isolates (*n* = 93) per facility and number of antibiotics (total *n* = 8) each isolate was resistant against. (**C**) Number of MDR and non-MDR *E. coli* isolates per facility. Facility 1, *n* = 58; Facility 2, *n* = 27; and Retirement, *n* = 8. FEP, cefepime; CAZ, ceftazidime; PIP-TAZ, piperacillin-tazobactam; CIP, ciprofloxacin; GEN, gentamicin; MER, meropenem; SXT, trimethoprim-sulfamethoxazole; COL, colistin; Ab(s), antibiotic(s).

Analysis of resistance was determined using percentages representing the relative proportion of isolates characteristic of each facility. This was carried out to mitigate the limitation associated with each facility housing a different number of residents. Analysis revealed that out of the 58 isolates from Facility 1 that were assessed, 49 (84.5 %) were found to be non-susceptible to the fourth-generation cephalosporin cefepime, and 57 (98.3 %) were non-susceptible to the third-generation cephalosporin ceftazidime These results contrast with those obtained for Facility 2, with only two out of the 27 (7.4 %) *E. coli* isolates displaying low to moderate levels of resistance against both cefepime (MICs of up to 2 mg/L) and ceftazidime (MICs of up to 16 mg/L) (Fig. 1A; Table 1).

MIC assays revealed high-level ciprofloxacin resistance of ≥64 mg/L (more than 1,024 times the ECOFF value) for 34.4% (*n* = 32/93) of all isolates recovered (Table 1). These isolates were relatively equally spread between the two facilities (43.1% and 25.9% of the total isolates from Facilities 1 and 2, respectively). High-level resistance was also observed against cefepime and ceftazidime with MICs of ≥64 mg/L seen in 12.9% (*n* = 12/93) and 39.8% (*n* = 37/93) of all isolates for cefepime and ceftazidime, respectively. High-level resistance against trimethoprim-sulfamethoxazole (>8 mg/L) was observed for 44 of the 93 isolates (Table 1). Almost all of those isolates (41/58) were from Facility 1 (Fig. 1A).

Facility 1 harbored a greater number of *E. coli* isolates resistant to multiple antibiotics (Fig. 1B). Facility 2 and Retirement *E. coli* isolates were observed to be primarily gentamicin and ciprofloxacin resistant. Although all isolates assessed in this study were resistant to at least one antibiotic, the MDR phenotype was primarily observed in *E. coli* isolates recovered from Facility 1 (Fig. 1B and C), with 54/58 (93.1 %) of the these shown to be resistant to three or more antimicrobial classes tested in this study (Fig. 1B and C). A much lower frequency (*n* = 5/27, 18.5%) of MDR *E. coli* were recovered in Facility 2, which was observed to harbor mostly non-MDR *E. coli* isolates.

The largest disparity observed between the isolates assessed in this study pertained to gentamicin resistance, with high levels of resistance (MICs of 64–128 mg/L) observed in 66.7% (*n* = 18/27) of Facility 2 *E. coli* isolates and only 8.6% (*n* = 5/58) of Facility 1 isolates. These findings indicate that initial selection using ciprofloxacin and ceftazidime did not limit the overall results obtained in this study and that the prevalence of resistant isolates was not determined by the number of occupants per facility. Finally, a low incidence of resistance was observed for both meropenem and colistin and was observed in isolates recovered from Facility 1 only (Fig. 1A).

**Fig 2:**
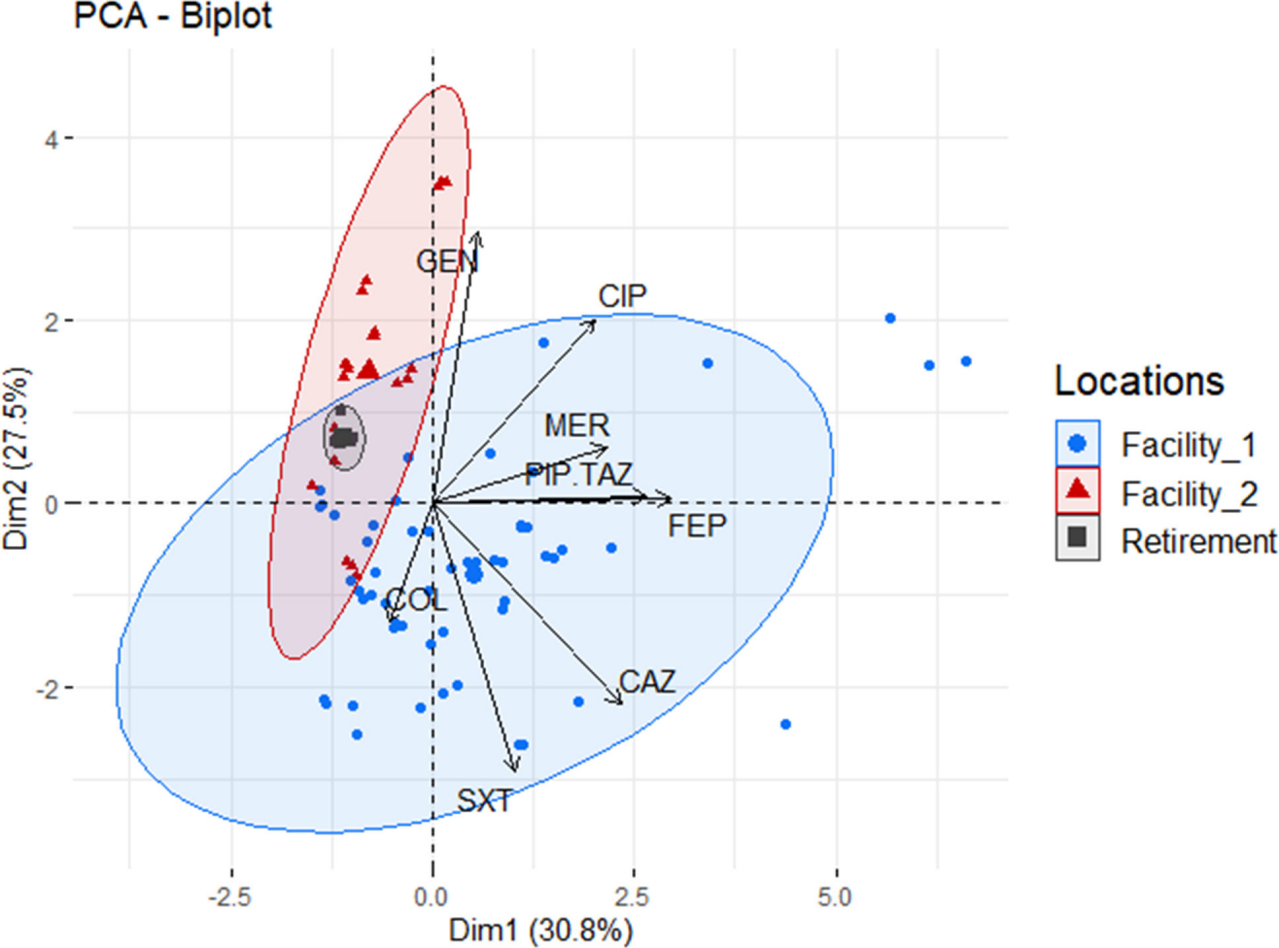
Principal component analysis (PCA) plot shows clustering of MDR *E. coli* isolates recovered from Facility 1 (blue) relative to isolates which are resistant to gentamicin (GEN) and ciprofloxacin (CIP) only and recovered from Facility 2 (red) and independent living facility (Retirement, gray). Ellipses were drawn at a confidence level of 0.95. CEP, cefepime; CTZ, ceftazidime; PIT, piperacillin-tazobactam; CIP, ciprofloxacin; GEN, gentamicin; MER, meropenem; TRS, trimethoprim-sulfamethoxazole; COL, colistin.

To further explore the distribution and association between MDR *E. coli* and isolation site, a PCA biplot was constructed (Fig. 2). As can be seen, the nature and direction of correlation between resistant isolates and facility indicate that Facility 1 clusters with isolates resistant to numerous antibiotics, as such displaying an MDR phenotype, while Facility 2 clustered with Retirement isolates displaying resistance to gentamicin and ciprofloxacin only. Unlike isolates recovered from the two RACFs, which spanned out across the two components, Retirement isolates clusters tightly and were grouped within the Facility 2 cluster. This clustering was expected as the resistance profiles for Facility 2 and Retirement isolates were comparable.

### Genomic analysis and isolate typing of a subset of AMR *E. coli*

Of the 93 wastewater *E. coli* isolates analyzed in this study, 35 were selected for WGS. Selection was based on their antimicrobial resistance profile and time of sample collection (Table S1). These were selected to further investigate genetic diversity and clonality of the isolates over the sampling period. As a greater number of MDR *E. coli* were isolated from Facility 1, these (*n* = 24/35, 68.6%) made up most of the samples sequenced. The remaining sequences included seven (20%) from Facility 2 and four (11.4%) from Retirement samples.

**Fig 3:**
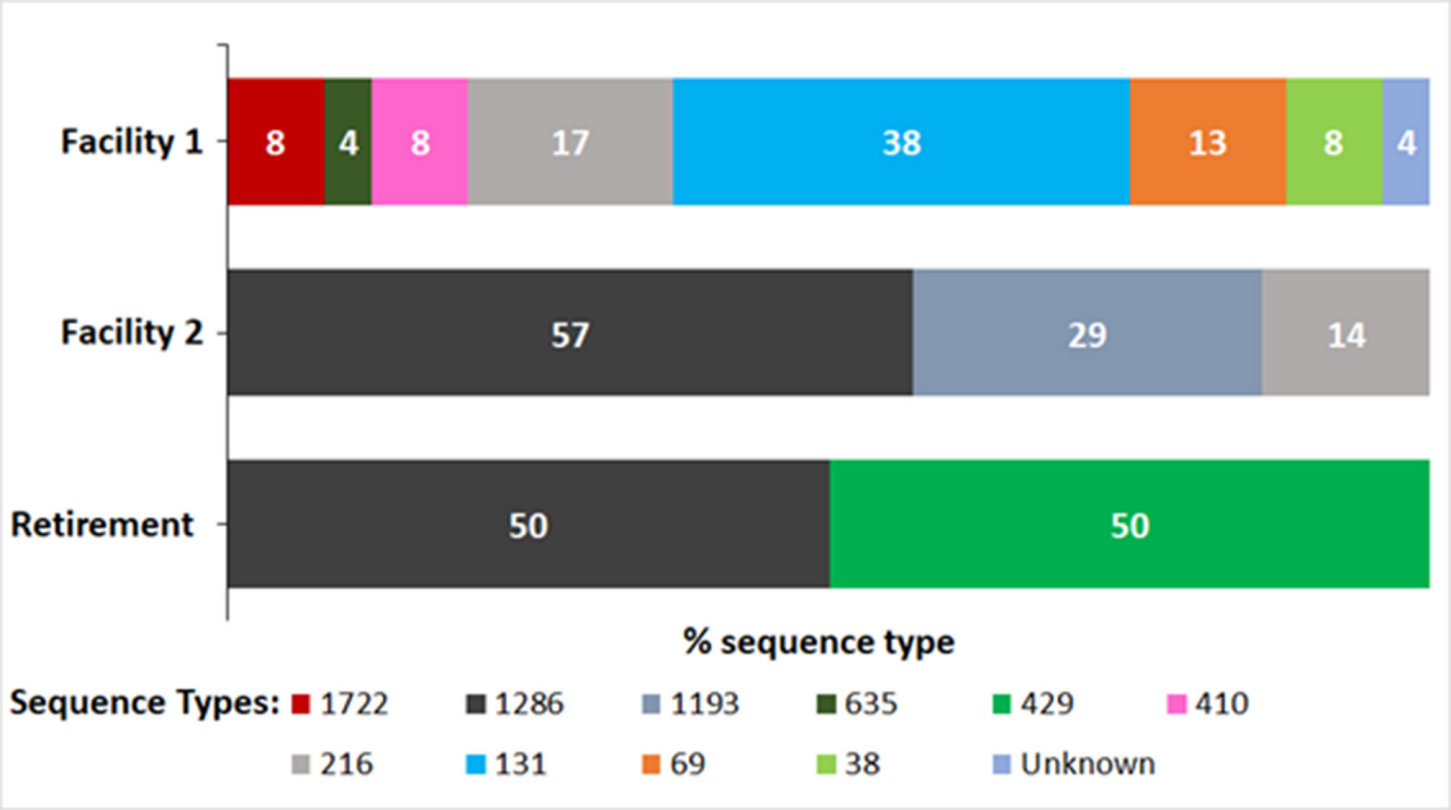
Distribution and percentage of sequenced *E. coli* isolate MLSTs per facility and retirement village. Facility 1, *n* = 24; Facility 2, *n* = 7; and Retirement, *n* = 4.

Genotyping and MLST analysis were used here to investigate the genetic diversity of the isolates sampled in this study. Results revealed 10 different sequence types (STs) and one unknown sequence type among the 35 sequenced *E. coli* isolates (Fig. 3). The pandemic ST131 was observed in samples isolated from Facility 1 and represented the largest ST (*n* = 9/24, 37.5%) of the isolates recovered from this facility. Moreover, two carbapenem-resistant *E. coli* isolates that belong to the international high-risk clone ST410 were detected in Facility 1. Facility 2 was represented by ST1286 (*n* = 4/7, 57.1%), which was also observed in Retirement isolates (*n* = 2/4, 50%). This sequence type is predominantly associated with environmental and animal isolates (64, 65) and, according to the EnteroBase database (https://enterobase.warwick.ac.uk/), has not been isolated previously in Australia. We observed two *E. coli* isolates of emerging pandemic clone ST1193 in Facility 2. This sequence type is the most predominant non-ST131 fluoroquinolone-resistant ST in the world (66).

**Fig 4:**
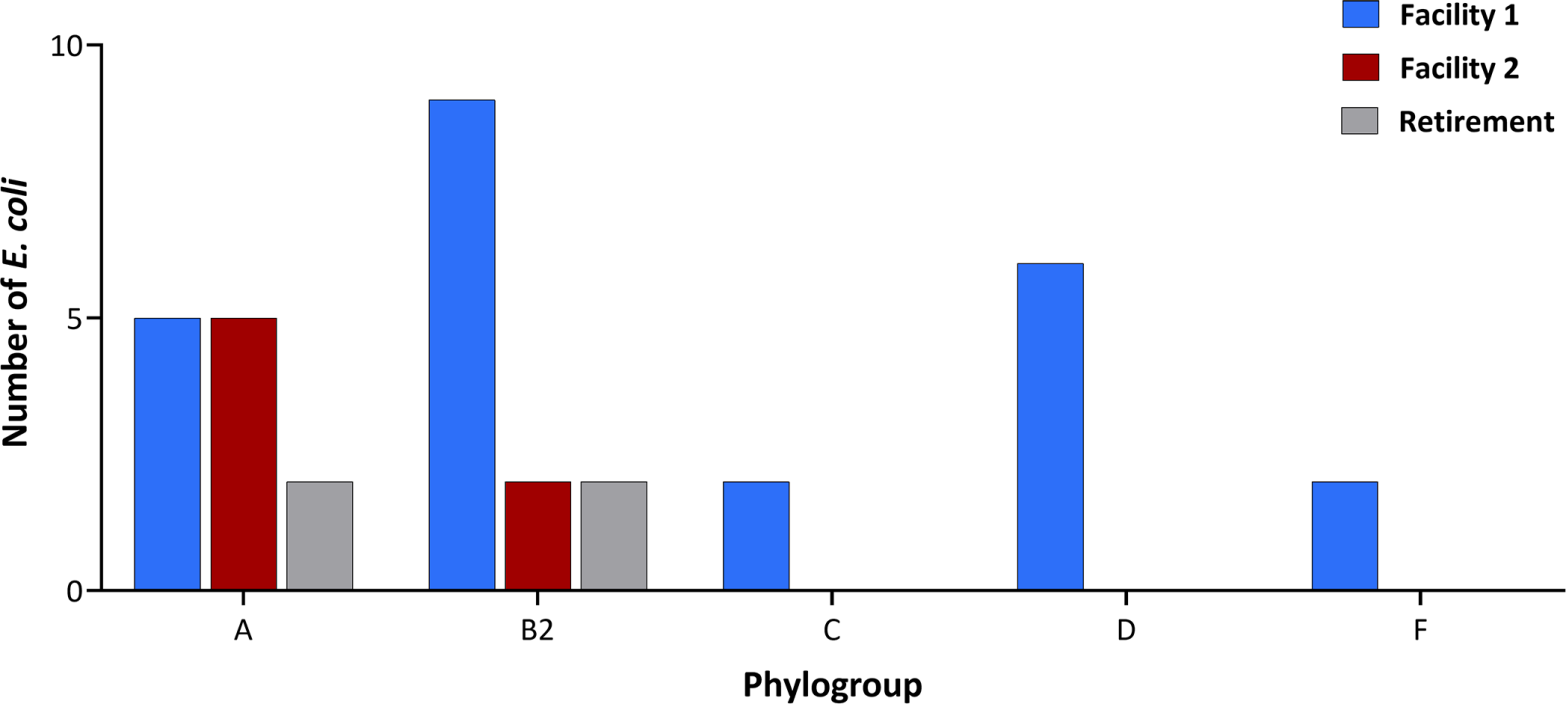
Phylogroup distribution of 35 *E. coli* isolates from two RACFs and a retirement site. Facility 1, *n* = 24; Facility 2, *n* = 7; and Retirement, *n* = 4.

To further type the sequenced isolates, phylogroup analysis was carried out based on the Clermont phylogroup typing method (51) (Fig. 4). Of the seven main phylogroups, A, B1, B2, C, D, E, and F (67), 37.1% (*n* = 13/35) of the sequenced *E. coli* isolates were classified to the increasingly MDR phylogroup B2. A higher prevalence (*n* = 9/35, 25.7%) of these isolates originated from Facility 1. Phylogroup A, representing commensal strains, was the second most prevalent phylogroup and observed with equal frequencies in Facilities 1 and 2 (both *n* = 5/35, 14.3%). Meanwhile, Retirement *E. coli* isolates were classified in to phylogroups A and B only, mirroring Facility 2 isolates. Of the three different sampling sites, Facility 1 isolates represented the greatest diversity in terms of phylogroups, with groups C, D, and F also assigned to these isolates. Strains assigned to group C are closely related to phylogroup B1 which represent commensal strains (68). Phylogroup D isolates, like those classified to group B2, are associated with virulent extra-intestinal *E. coli* infections (69). Strains assigned to phylogroup F have been found to be ESBL producing and resistant to fluoroquinolones (70).

**Fig 5:**
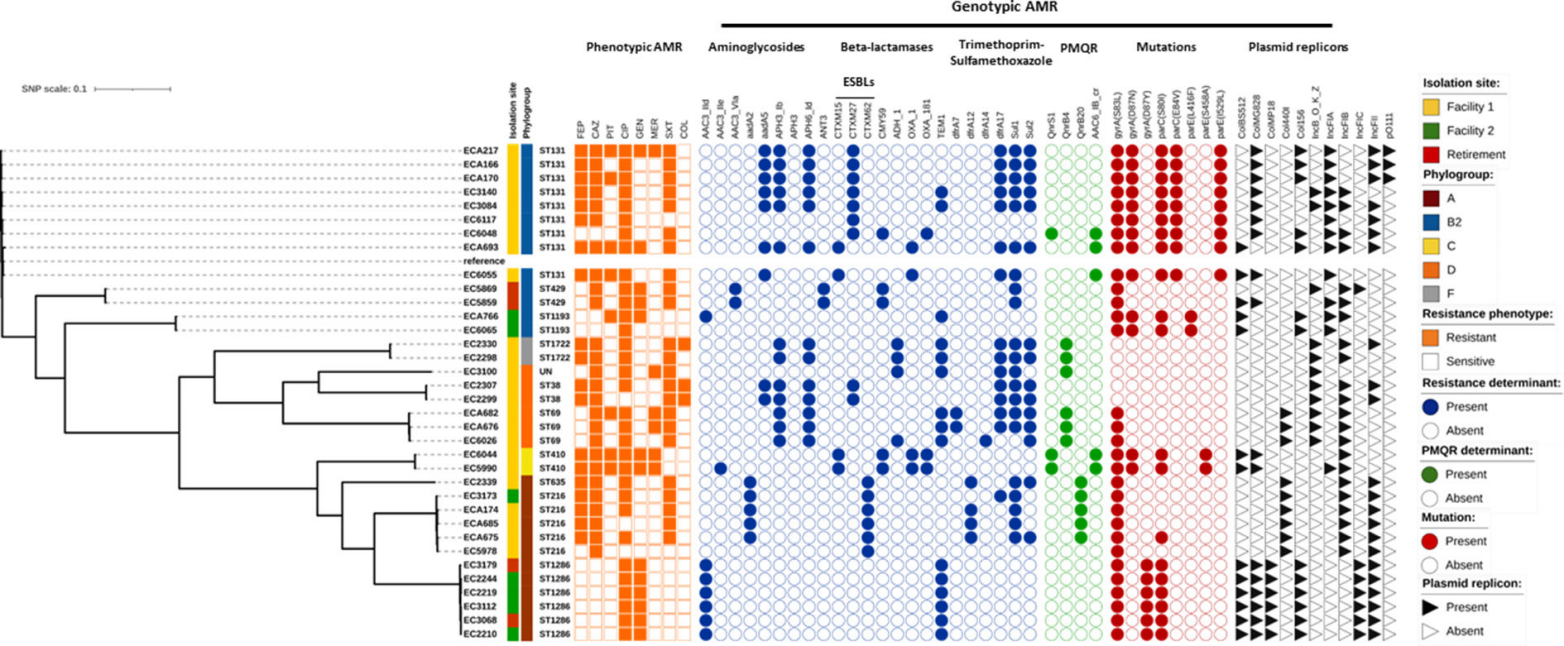
Phylogenetic SNP analysis of 35 AMR *E. coli* isolated from RACFs and a retirement Village in South Australia, corresponding resistance profile presence of AMR determinants and plasmid replicons. SNP analysis was performed using the isolate EC958 (accession number: NZ_HG941718.1) as the reference genome. Vertical columns demonstrate (1) isolation site, (2) phylogroups, (3) resistance phenotype: resistant (filled squares) and sensitive (empty squares), (4) resistance genotype including plasmid-mediated quinolone resistance (PMQR): presence (filled circles) and absence (empty circles) of acquired resistance genes, (5) mutations in the quinolone resistance-determining regions: present (blue filled rectangle) and absent (empty rectangle), (7) predominant plasmid replicon types: presence (filled left side triangles) and absence (empty left side triangles). FEP, cefepime; CAZ, ceftazidime; PIT, piperacillin-tazobactam; CIP, ciprofloxacin; GEN, gentamicin; MER, meropenem; SXT, trimethoprim-sulfamethoxazole; COL, colistin.

Finally, core genome SNP analysis was used to assess clonal relatedness of all 35 sequenced *E. coli* isolates, with the results represented in a maximum-likelihood phylogenetic tree (Fig. 5). Isolates were aligned to *E. coli* EC958 (accession number: NZ_HG941718.1), which represents an international MDR strain. Analysis revealed a clustering of isolates by facility, sequence type, susceptibility profile, and phylogroup indicating clonality. Nonetheless, variability between AMR determinants and plasmid replicons was also observed between clonally related isolates.

### Identification of *E. coli* AMR genes

Given the observations that the wastewater isolates assessed in this study displayed a similar resistance profile per facility, we sought to evaluate their genotypic diversity and examined their genomes for the presence of mobile AMR genes and potential plasmids. All these wastewater isolates were shown to possess a plethora of AMR genes, contributing both to their intrinsic and acquired resistance. Twenty-seven different AMR genes conferring resistance to the compounds assessed in this study, which included cephalosporins, carbapenems, β-lactam- β-lactam-inhibitor combinations, fluoroquinolones, aminoglycosides, trimethoprim, and sulphonamides, were identified (Fig. 5). The extended-spectrum beta-lactamases (ESBLs) such as the *bla*_CTX-M_ type genes accounted for 37.3% (*n* = 19/51) of the beta-lactamase genes detected in the wastewater samples. The *bla*_CTX-M_ type genes (*n* = 19) included *bla*_CTX-M-15_ (*n* = 4/19, 21.1%), *bla*_CTX-M-27_ (*n* = 9/19, 47.4%), and *bla*_CTX-M-62_ (*n* = 6/19, 31.6%). Except for one *bla*_CTX-M-62_ carrying strain isolated from Facility 2, all other *bla*_CTX-M_ type genes were found in Facility 1 isolates. Non-ESBL beta-lactamases, *bla*_OXA-1_ (*n* = 4/35, 11.4%) and *bla*_OXA-181_ (*n* = 2/35, 5.7%), were identified in isolates recovered from Facility 1 only, whereas *bla*_TEM-1_ was detected in *E. coli* isolated from Facility 1 (*n* = 8/35, 22.9%), Facility 2 (*n* = 5/35, 14.3%), and retirement (*n* = 2/35, 5.7%). No carbapenemase genes were detected despite meropenem resistance in four out of the six sequenced meropenem-resistant *E. coli* isolates (Fig. 5). However, these isolates harbored at least one ESBL or AmpC beta-lactamase genes. Non-carbapenemase-producing low-level carbapenem resistance in *E. coli* could be mediated by ESBL or AmpC beta-lactamases associated with an overexpression of efflux pumps (such as AcrAB) or a loss of porin (OmpF) expression (71, 72).

Bacteria with ESBL phenotypes are frequently found to harbor additional resistance genes. In this study, isolates were found to carry the plasmid-mediated quinolone resistance (PMQR) genes, which were detected in Facility 1 isolated strains only (*n* = 14/35, 40%). Of these, the most prevalent PMQR gene detected was *qnrB*4 (*n* = 6/14, 42.9%). Also only isolated from Facility 1 was the ciprofloxacin and aminoglycoside modifying enzyme (*aac-(6’)-lb-cr),* accounting for 14.3% (*n* = 5/35) of the total isolates detected. Genes mediating aminoglycoside resistance such as *aac(3)-IId* were also detected in 7 out of 35 isolates (20%) and exclusively recovered from Facility 2 and Retirement, whereas *aph3Ib* and *aph6Id* (both *n* = 13/35, 37.1%)*,* which are commonly found on plasmids, integrative elements, and chromosomal islands, were detected in wastewater isolates recovered from Facility 1. A higher prevalence of the sulfonamide genes, *sul1* (*n* = 21/35, 60%) and *sul2* (*n* = 15/35, 42.9%), and trimethoprim *dfrA17* (*n* = 15/35, 42.9%) resistance genes were also observed, with the majority detected in Facility 1 isolates. Lastly, ciprofloxacin resistance due to point mutations was also investigated, with 32/35 (91.4 %) isolates shown to be phenotypically ciprofloxacin resistant. Of these, 31/32 (96.9 %) were shown to possess mutations within *gyrA, parC*, or *parE* genes (Table S2).

### Prevalence of plasmid replicon types detected in AMR *E. coli* isolates

Plasmids are known to carry and distribute AMR genes such as ESBLs, carbapenemases, and PMQR genes (73). Due to their ability to transmit AMR genes to other bacteria, they can enable the rapid evolution of bacteria in response to different environmental pressures (74). In this study, analysis of plasmids within the 35 sequenced wastewater *E. coli* isolates were differentiated based on plasmid replicon types. The incompatibility groups (Inc) IncFIB (*n* = 22/35, 62.9%) and IncFII (*n* = 22/35, 62.9%) accounted for the prevalent replicon types detected in the *E. coli* isolates. All isolates were positive for at least one Inc or replicate (Rep) group. Analysis of the most dominant plasmid replicon type by facility revealed Facility 1 isolates to harbor IncF (IncFIA, IncFIB, IncFIC, and IncFII) type plasmids, Facility 2 Col (ColBS512, ColMG828, ColMP18, Col440I, and Col156) type plasmids, with Retirement isolates harboring both types with near-equal distribution.

### Relative abundance of bacterial families represented by metagenomics

**Fig 6:**
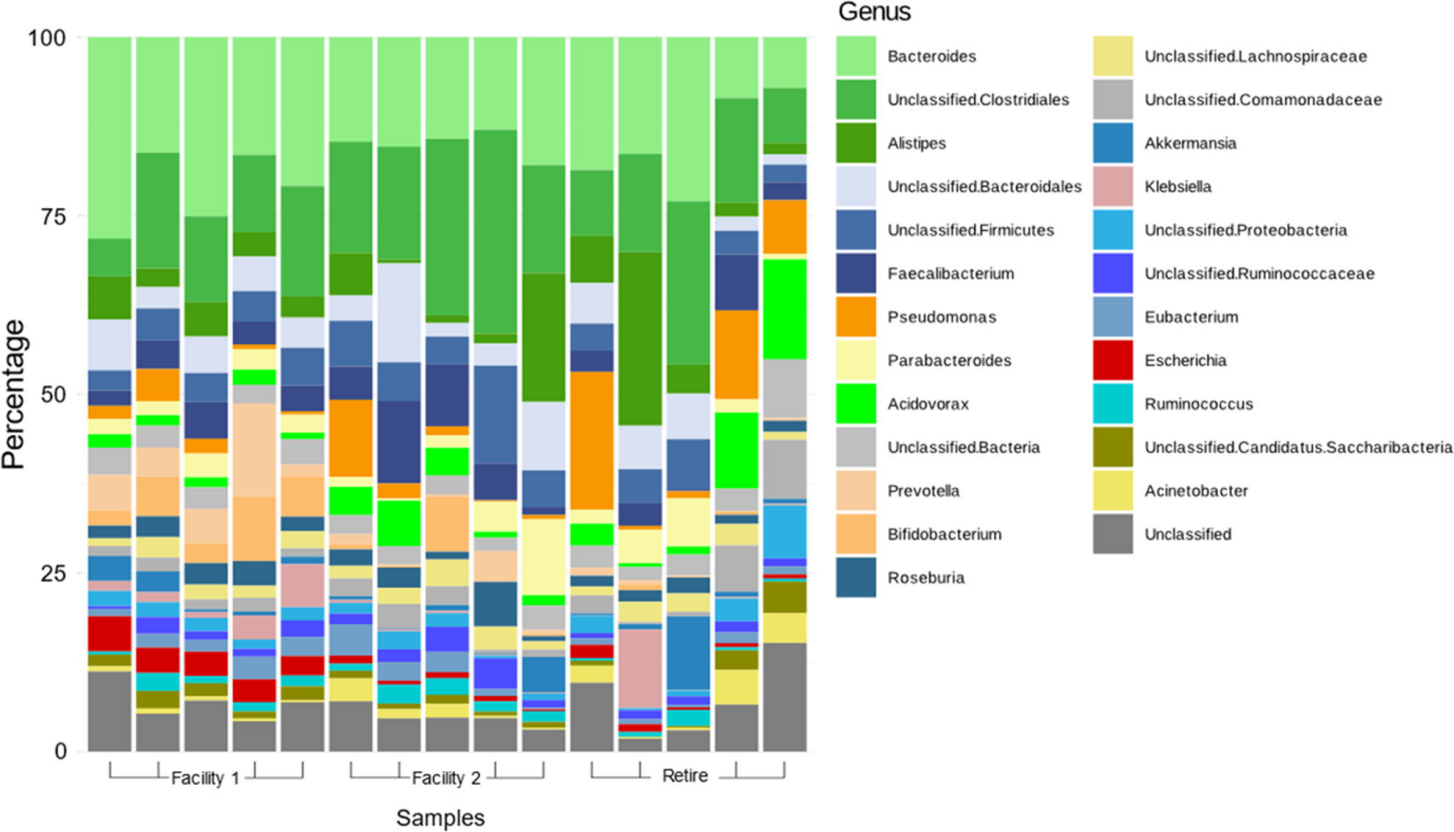
Relative abundance (%) of the top 25 genera identified in wastewater collected from two RACFs and retirement samples. Red bars represent bacteria belonging to the *Escherichia* genus.

To compare the complete wastewater resistome with data obtained from the *E. coli* isolates, metagenomic sequences were obtained from all sample sites and used here to assess taxonomy and prevalence of AMR in each site. Taxonomic annotation of the metagenomic data sets revealed the 25 most abundant genera detected in Facility 1, 2, and Retirement wastewater samples (Fig. 6). Bacteria belonging to the *Escherichia* genus represented an average of 3.6% of bacteria in Facility 1, 0.7% Facility 2, and 0.9 % in Retirement. A comparison between Facility 2 and Retirement revealed a greater number of AMR *E. coli* isolates recovered from Facility 2 despite Retirement presenting an overall greater abundance of these organisms. This exemplifies the importance of culturing in the assessment of prevalence of AMR or MDR organisms within wastewater samples.

### Occurrence and abundance of AMR genes in wastewater samples

Given the phenotypic-genotypic relationship in *E. coli* (75 – 77), this study focused on acquired resistance genes, which, as a result of horizontal gene exchange, have rendered many important pathogens such as MDR *E. coli* potentially unresponsive to current treatment. The focus of this study included the ESBL *bla*_CTX-M_, beta-lactamases *bla*_OXA_, *bla*_TEM_ and *bla*_IMP_, the dihydrofolate reductase genes *dfrA1*, and the dihydropteroate synthases *sul1* and *sul2*, which confer bacterial resistance against trimethoprim and sulfonamide. In addition to these, PMQR genes *qnrB* and *qnr*S and *aac(6′)-Ib-cr* (cr for ciprofloxacin resistance) were also evaluated. Finally, prevalence of gentamicin resistance was explored by the assessment of the *aac(3)* and *aac(6′*) genes.

**Fig 7:**
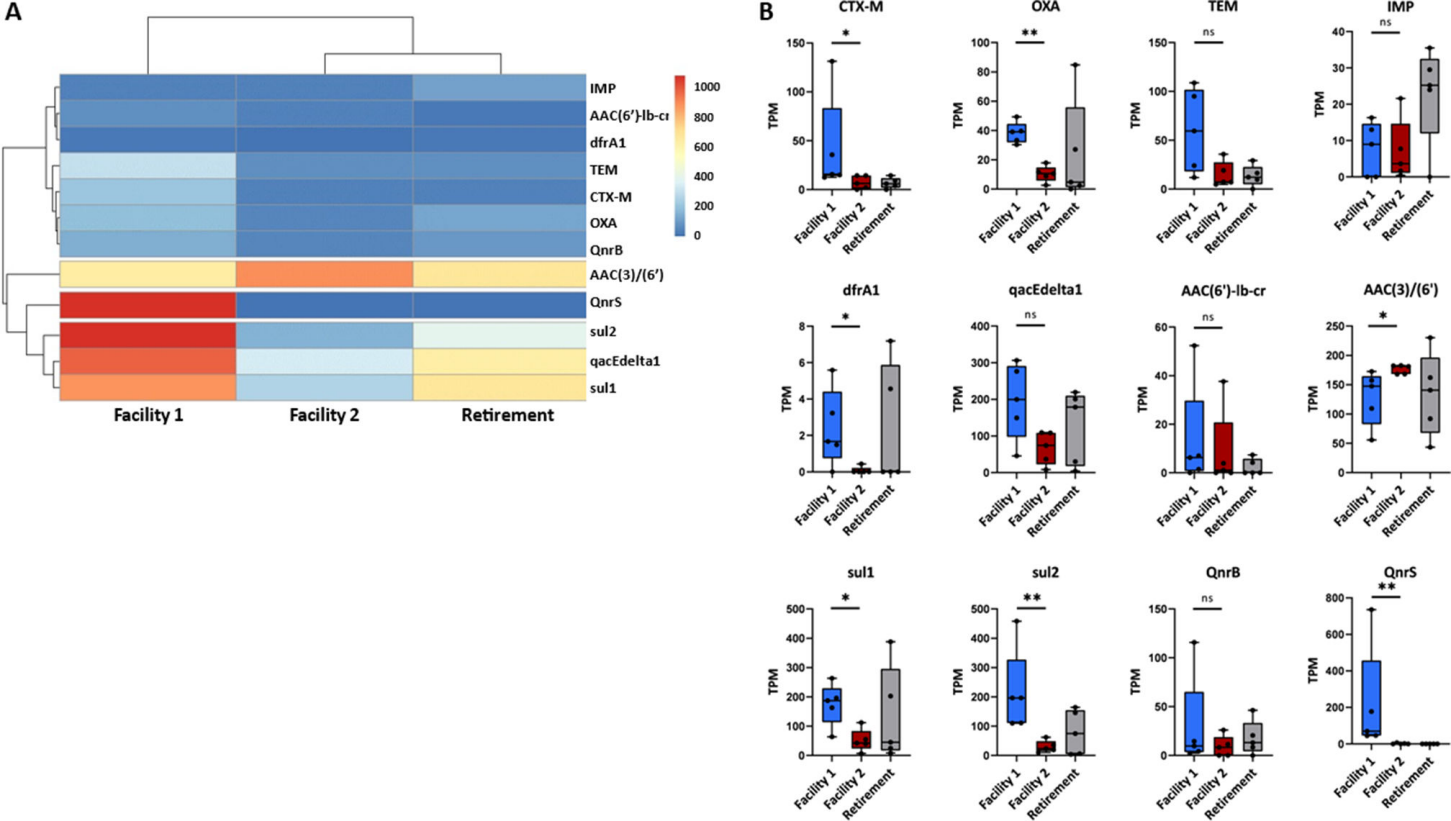
Distribution of mobile resistance genes in wastewater from RACFs and an independent living facility (retirement). (**A**) Heatmap of distribution and abundance clustering of resistance genes. AMR genes are shown on the right vertical axis. Hierarchical clustering was carried out on both resistance genes and sites. AMR abundance is normalized as TMP (transcripts per million reads). (**B**) Boxplots of select AMR gene reads per ten million (TPM) per facility. Each sample is represented by a dot with horizontal jitter for visibility. The horizontal box lines represent the first quartile, the median, and the third quartile. Whiskers represent the range of points within the first quartile −1.5 × the interquartile range and the third quartile +1.5 × the interquartile range. Differences were considered statistically significant at *P < 0.05 and **P < 0.01 level, determined by a two-tailed Mann-Whitney U test.

A higher abundance of most of the acquired resistance genes assessed in this study, by a comparison of TPM values obtained for the AMR genes, was detected in samples originating from Facility 1 (Fig. 7A). The exception to this were Facility 2 samples which had a larger proportion of the gentamicin resistance genes *aac(3)* and *aac(6’*) and Retirement samples which had a higher prevalence of the beta-lactamase *bla*_IMP_. The differences between Facilities 1 and 2 samples regarding the number of the different AMR genes assessed here were determined to be significant (*P* < 0.05; Fig. 7B). Differences were seen in beta-lactamase *bla*_CTX-M_, *bla*_OXA_ genes, the dihydrofolate reductase *dfrA1* genes, the 3-N-aminoglycoside acetyltransferase *aac(3*), and the 6′-N-aminoglycoside acetyltransferase *aac(6’*) genes, as well as the sulfonamide *sul1* and *sul2* genes and the quinolone antibiotic resistance genes *qnrS*. These results clearly show that Facility 2 has a lower relative abundance of the mobile AMR genes assessed in this study, all of which have been associated with human pathogens. No significant differences were found for the AMR abundance between Facility 2 and Retirement samples.

## DISCUSSION

The rise of AMR and emergence of bacteria that are resistant to multiple classes of antibiotics presents a global threat to human health (78). Measures taken to fight resistance include broadening our understanding of the selective pressures driving AMR to be able to mitigate them. RACFs have been identified as sites harboring elevated levels of AMR bacteria and presenting an environment which is highly selective to their development and emergence (79, 80).

In this study, the prevalence of AMR *E. coli* was used as an indicator of the incidence and persistence of bacterial resistance in two RACFs, one of which has implemented an AMS program (Facility 2), while the other has not (Facility 1). Assessment of the prevalence of *E. coli* in these two RACFs and in one retirement village was carried out with an in-depth analysis of 93 AMR *E. coli* isolates recovered from wastewater samples, collected over five different time points. Results revealed a high proportion of MDR *E. coli* isolates (66.7%) which were more frequently recovered from Facility 1. Isolates recovered from Facility 1 displayed high levels of resistance against ceftazidime, cefepime, and ciprofloxacin with MIC values of >128, >512, and >1024 times above the ECOFF. While Facility 2 isolates displayed high-level gentamicin resistance, with MIC values of >64 mg/L (>32 times above the ECOFF), a trend not observed in Facility 1 *E. coli* isolates. These results highlight several concerns, including the high prevalence of ESBL-producing *E. coli* in Facility 1 and the number of ciprofloxacin-resistant *E. coli* isolates prevalent in both facilities.

According to the Antimicrobial Use and Resistance in Australia (AURA) Surveillance System (2019), rates of resistance for many priority organisms, including *E. coli*, have not changed significantly over the last 2 years. However, there are several exceptions to this, including increasing rates of resistance observed in *E. coli* against commonly used agents such as ceftriaxone, a third-generation cephalosporin and ciprofloxacin, among other fluoroquinolones, despite restricted access in the community and guidelines advising these not to be prescribed when other options are available, and if needed to be prescribed for the shortest duration clinically possible (AURA, 2019). Of the two cephalosporins assessed here, higher rates (66.7%) of resistance were seen for the third-generation ceftazidime than for the fourth-generation extended-spectrum agent, cefepime (53.7% of all isolates). Whole genome sequencing of ESBL-producing *E. coli* revealed the presence of CTX-M-type genes assigned to ST131 isolates. This sequence type is a major contributor to both sepsis and urinary tract infections (UTIs) in Australia (81). Moreover, phylo-typing assigned these isolates to the B2 phylogenetic group. This group is frequently associated with CTX-M-15-producing *E. coli* (82), although, in this study, it was associated with CTX-M-27-producing isolates. Globally, CTX-M-15-producing strains are recognized as the main drivers of the pandemic spread of this sequence type (83). However, genomic comparisons of ST131 isolates have shown that both CTX-M-15- and CTX-M-27-producing isolates dominate in Australia (81). CTX-M-27 ESBLs were also initially postulated to be confined to ST131 (84); however, these have now also been identified in ST38 *E. coli* isolates, as also observed in this study, suggesting an emergence of new ESBL-producing clones (85). The dissemination and continual emergence of these ESBLs and others are facilitated by their presence on plasmids belonging to the IncF group (86), among others, which were also detected here. This plasmid group is often associated with *bla*_CTX-M-15_, *bla*_TEM-1_, *bla*_OXA-1_, and *aac(6′)lb-cr* resistance genes, all of which were detected here and largely in isolates recovered from Facility 1.

The isolation of ST131 in this study highlights the continual spread of this pandemic clonal group. Although a worrying trend, the presence of ST131 *E. coli* isolates within RACFs has been observed worldwide (87, 88). It has been postulated that isolates of this sequence type have superior transmission and colonization abilities, allowing for rapid global proliferation (89 – 91). These characteristics coupled with the intrinsic nature of RACFs and residents therein make the presence of this clonal group within RACFs a significant concern.

In addition to being resistant to extended-spectrum cephalosporins, ST131 *E. coli* clones are also associated with fluroquinolone resistance (92) which can be coupled with aminoglycoside and trimethoprim-sulfamethoxazole resistance (90). In this study, all ST131 isolates were ciprofloxacin resistant and, except for one isolate, trimethoprim-sulfamethoxazole resistant. Worryingly, two isolates were extensively drug resistant with resistance against all the tested classes of antibiotics observed. Fluroquinolone resistance continues to be of concern and was observed in this study with high levels of ciprofloxacin resistance (MICs of >64 mg/L) detected in isolates recovered from both facilities and in retirement samples. According to the Australian Group of Antimicrobial Resistance Sepsis Outcome Programs, 2021, rates of fluroquinolone-resistant *E. coli* have continued to increase from 13.7% in 2013 to 21.8 % in 2021. Increasing trends have also been observed in Europe with the European Antimicrobial Resistance Surveillance Network reporting 25.3% fluoroquinolone-resistant *E. coli* isolated in 2018, representing an increase from previous years (93). This upward trend is also reported in *E. coli* isolated in Asia as well as North America (94). Various theories have been presented regarding the acquisition of fluoroquinolone-resistant *E. coli* including possible colonization with these as a result of hospital admissions (95), the continual use of these in treatment of UTIs (96), and clonal spread (97).

Analysis of fluoroquinolone resistance genes within WGS isolates revealed the presence of numerous ciprofloxacin resistance genes including the transferable PMQR determinants *qnr* and *aac(6)-Ib-cr*. The presence of these is of concern as these are able to be transferred to other bacteria, are often associated with elements such as insertion sequences, transposons, phages, and plasmids which enhances their transferral, and can be accompanied by other mobile resistance genes (98).

Metagenomic analysis of the prevalence of these and other mobile resistance genes within the sampled wastewater revealed that Facility 1 harbored a greater number of mobile resistance determinants. A limitation of using the metagenomic method for the assessment of bacterial and AMR gene abundance is that each sample of wastewater represents a single point in time. As such, wastewater components may be influenced by the amount of fecal matter in the samples and the activities being performed at the time of collection, such as cleaning or washing and the detergents being used, all of which may alter the microbial and antimicrobial gene abundance within each sample. To minimize this limitation, sites were sampled over a 10-hour period at five different time points throughout an 18-month period, and samples were assessed by microbial culturing and metagenomics. To ensure that the data are not influenced by the number of people, flow rate at the time points of grab sample collection, or any other potential variables, the data obtained from metagenomics was normalized, and AMR gene abundance was calculated as transcripts per million mapped bacterial reads. In addition to Facility 1 samples, which displayed consistently higher levels of bacteria belonging to the *Escherichia* genus, metagenomic results also revealed a higher prevalence of these in Retirement wastewater samples when compared to Facility 2 but an overall lower prevalence of AMR *E. coli* upon culturing. As such, the higher prevalence of AMR *E. coli*, as identified by microbial culturing and metagenomics for all five sampling periods, is indicative of a general trend only seen for Facility 1. This trend was also observed with respect to mobile resistance genes, with Facility 1 displaying a comparatively higher abundance of certain resistance genes.

Analysis of the mobile resistance genes revealed plasmid-mediated resistance genes, some of which are associated with Class 1 integrons that have been isolated from AMR clinical isolates belonging to both the Enterobacteriaceae and Pseudomonaceae families (99). Having the ability to integrate gene cassettes which confer resistance to a broad range of antibiotics has made the prevalence of Class 1 integrons a proxy for bacterial resistance (100). As such, the greater presence of the mobile resistance genes may be indicative of a higher presence of AMR bacteria in Facility 1 wastewater.

Limitations of this study include that only two RACFs were assessed and compared and that these differed in the number of residents residing in them. However, all variables that were reported in this study were the same between the facilities, for example, the age of the cohort and type of care provided. Moreover, the two facilities were managed by the same age care provider, which runs additional facilities in Australia. It is possible that there could have been differences in variables that were not measured as part of this study such as the socio-economic status of the demographic, duration of stay, staff turnover number, or age of the facility. To overcome variations in resident numbers and isolate prevalence, percentages were utilized for standardized comparisons, allowing meaningful assessments across facilities in the study. However, to our knowledge, this is the first study to include such a comprehensive, all-encompassing testing protocol. This study has included isolation of *E. coli* and culture-based genotypic AMR analysis, genomic analysis of the resistome of individual organisms, and metagenomic sequencing and analysis of the total wastewater resistome. It is also the first time that a direct comparison of two RACFs has been undertaken, with a spotlight on AMR.

The findings of this study offer an insight into bacterial resistance and the prevalence thereof within RACFs. Here, one facility was found to harbor higher rates of AMR *E. coli,* including the pandemic clone ST131. Moreover, metagenomic analysis revealed a higher incidence of mobile resistance determinants in wastewater sourced from this facility. The inclusion of microbial culturing in combination with whole genome sequencing and metagenomic analysis has allowed for a more complete assessment of the RACFs evaluated in this study. Based on the differences in the prevalence of AMR *E. coli* and mobile resistance genes within the RACFs assessed here, our results show that each facility presents a unique environment which may select for resistance and possible transmission of AMR bacteria. Mitigating this may be the implementation of an AMS program, which was present in Facility 2. However, it is understandable that the implementation of AMS programs in RACFs can be complicated and is dependent on numerous factors including access to resources, staff education and a high prevalence of bacterial colonization and challenges in diagnosing and treating infections (101). Nonetheless, the benefits of such programs can help mitigate the spread and continual development of AMR.

Currently, there are significant gaps in the surveillance of AMR and MDR organisms within RACFs. Our understanding of the colonization of such organisms in RACF residents is also lacking, as is knowledge pertaining to the risk factors which aid both the development of AMR and potential spread of both AMR and MDR organisms and high-risk antimicrobial genes from RACFs to the wider community. The results generated in this study highlight the need for ongoing surveillance studies focusing on RACFs, which, given our aging population, are greatly needed to address and possibly mitigate the ongoing threat of AMR.

## Data Availability

Whole genome and shotgun metagenomic sequences were deposited in the NCBI database under Bio Project number PRJNA861152. Reads mapping to the human reference genome (GCA_000001405.15) were removed prior to submission to public sequence databases according to the protocol used in the Human Microbiome Project (102, 103).

## SUPPLEMENTAL MATERIAL

File S1 spectrum.00731-23-s0001.docx Antimicrobial stewardship (AMS) strategies followed by Facility 2.

Table S1 spectrum.00731-23-s0002.xlsx Overview of antibiotic resistance profiles.

Table S2 spectrum.00731-23-s0003.xlsx GyrA, ParC, and ParE mutations in 35 ciprofloxacin resistant *E. coli* isolates.

ASM does not own the copyrights to Supplemental Material that may be linked to, or accessed through, an article. The authors have granted ASM a non-exclusive, world-wide license to publish the Supplemental Material files. Please contact the corresponding author directly for reuse.
