# Supplementary material for: The scope of antimicrobial resistance in residential aged care facilities determined through analysis of Escherichia coli and the total wastewater resistome
Source: Microbiol Spectr. 2023 Oct 3;11(6):e00731-23. doi: 10.1128/spectrum.00731-23 (PMC10715142; doi:10.1128/spectrum.00731-23)
Supplement: File S1 — Antimicrobial stewardship (AMS) strategies followed by Facility 2. [file spectrum.00731-23-s0001.docx]

**Supplemental 1:** Antimicrobial stewardship (AMS) strategies followed by Facility 2.

**Essential AMS strategies as listed by the Australian Commission on Safety and Quality in Health Care:**

**Implementing clinical guidelines which are consistent with the Therapeutic Guidelines: Antibiotic that take into account local microbiology and antimicrobial susceptibility patterns**

Has this strategy been implemented in this facility as part of the AMS program? Yes:  No:

**Restricting broad-spectrum and later-generation antimicrobials to residents in whom their use is clinically justified**

Has this strategy been implemented in this facility as part of the AMS program? Yes:  No:

**Reviewing antimicrobial prescribing, with intervention and providing direct feedback to the prescriber/doctor**

Has this strategy been implemented in this facility as part of the AMS program? Yes:  No:

**Using directed therapies, intravenous-to-oral switching and dose optimisation**

Has this strategy been implemented in this facility as part of the AMS program? Yes:  No:

**Ensuring that the clinical microbiology service used by the facility to assess infections**

–provides guidance and support for optimal specimen collection Yes:  No:

–targets reporting of clinically meaningful pathogens and their susceptibilities Yes:  No:

–uses selective reporting of susceptibility testing results Yes:  No:

–generates location-specific antimicrobial susceptibility reports (antibiograms) annually Yes:  No:

Has this strategy been implemented in this facility as part of the AMS program? Yes:  No:

**Monitoring antimicrobial use and outcomes and reporting to clinicians and management**

Has this strategy been implemented in this facility as part of the AMS program? Yes:  No:

**--------------------------------------------------------------------------------------------------------------------------------------**

***Nurses have been identified to play a key role in combating antimicrobial resistance, with the following identified as key strategies undertaken by nurses as part of AMS –were these implemented by this facility as part of the AMS program?***

Helping to reduce unnecessary and inappropriate antibiotic use

Promoting and practising good infection control

Educating patients and the general public on the issue of AMR and the things that people can do to prevent it getting worse

***Staff (prescriber, nurse and healthcare workers) education is also vital and includes the below listed actions – were these implemented as part of the AMS program?***

Promoting and practising standard prevention precautions including hand washing and other infection control measures

Recognising signs and symptoms of infection

Initiating clinical assessment and review

Ensuring that timely specimens for microbiology are collected correctly

Promoting the use of prescribing guidelines

Providing information and support to patients and their families about their health condition, treatment options, and how and why to take medicines according to the doctor’s instructions

Medication administration and management

Ensuring treatments are recorded properly in the patient’s health record.
